# Clinical significance of the UGT1A1*28 allele detection in HIV-infected patients

**DOI:** 10.7448/IAS.17.4.19579

**Published:** 2014-11-02

**Authors:** Veronika Kanestri, Konstantin Mironov, Alexey Kravchenko, Anastasiya Pokrovskaya, Olga Dribnohodova, Elena Dunayeva, Galina Tsiganova, Marina Harbutly, Marina Goliusova, Vladislav Konnov, Nadezhda Kozirina, Vasiliy Shahgildyan, Ulyana Kuimova, Anna Popova, Oksana Efremova, Danila Konnov

**Affiliations:** 1 Central Scientific Research Institute of Epidemiology, Russian AIDS Federal Center, Moscow, Russian Federation; 2 Department of Genetic Polimorphisms, Central Scientific Research Institute of Epidemiology, Moscow, Russian Federation

## Abstract

**Introduction:**

The UGT1A1*28 (rs8175347) polymorphism is associated with hyperbilirubinemia. The presence of 6 TA-repeats in the UGT1A1 gene promoter region corresponds to normal UGT1TA1 activity. A detection of 7 TA-repeats in hetero- or homozygous individuals [(TA)6/(TA)7 and (TA)7/(TA)7] is associated with lower UGT1TA1 activity, which may eventually result in the development of Gilbert syndrome and/or modified individual response to drugs metabolized by this enzyme. ATV contributes to the decreased levels of UGT1A1, which may lead to elevations of indirect bilirubin, jaundice and even to therapy discontinuation. We evaluated the prevalence of the UGT1A1*28 among HIV-infected patients and the dependence of the frequency and severity of AE during ATV treatment on individual genetic characteristics.

**Materials and Methods:**

47 HIV-infected patients was screen for UGT1A1 genotype and the presence of UGT1A1*28. All patients received ATV in the HAART regimen for 48 weeks. Changes in the total, direct and indirect bilirubin, ALT, AST, GGT and jaundice were evaluated. Statistical analysis was performed using Microsoft Office Excel for Windows XP Professional 2007 and Biostat.

**Results:**

All patients were followed up in the AIDS Center (males 72.3%, median age 33 years, median CD4+ count-282 cells/µl (19.5%)). HBV/HCV was in 36.2% patients. Ten patients had risk factors that could affect bilirubin turnover (chronic cholecystitis, biliary dyskinesia, etc.). Genotype (TA)6/(TA)6 was found in 42.6% patients, (TA)6/(TA)7-42.6% and (TA)7/(TA)7-14.9%. Overall prevalence of UGT1A1*28 was 57.4%, and homozygous allele frequency was 14.9%. G3/4 of indirect bilirubin were detected in 36.2% patients [(TA)6/(TA)6 in 10–20%, (TA)6/(TA)7-25-40%, (TA)7/(TA)7-72-86%], and significant jaundice in 10.6% [80% with (TA)7/(TA)7]. The OR for hyperbilirubinemia>40 µmol/L in patients with heterozygous UGT1A1*28 was increased 3 times over patients without this allele (OR 3.07, 95% CI 1.54–4.6) and 34 times as compared with homozygotes (OR 33.9, 95% CI 31.45–36.35). The presence of additional risk factors increased the probability of G3/4 hyperbilirubinemia. No significant changes in the ALT, AST, and GGT levels were observed.

**Conclusions:**

The risk of severe hyperbilirubinemia during ATV treatment is minimal for patients without UGT1A1*28 and no more than one additional risk factor and for patients with UGT1A1*28 and no additional risk factors; patients with homozygous genotype UGT1A1*28 are at the highest risk.
